# Supplementary material for: Navigating cross-reactivity and host species effects in a serological assay: A case study of the microscopic agglutination test for Leptospira serology
Source: PLoS Negl Trop Dis. 2024 Oct 4;18(10):e0012042. doi: 10.1371/journal.pntd.0012042 (PMC11482713; doi:10.1371/journal.pntd.0012042)
Supplement: Attachment — Submitted filename: Mummah_Navigating cross-reactivity_PLOS_ReviewerResponse.docx [file pntd.0012042.s002.docx]

**Title:** Navigating cross-reactivity and host species effects in a serological assay: A case study of the microscopic agglutination test for Leptospira serology

**Journal:** PLOS Neglected Tropical Diseases

Dear Editors:

Thank you for the thorough and thoughtful reviews of our manuscript titled “Navigating cross-reactivity and host species effects in a serological assay: A case study of the microscopic agglutination test for Leptospira serology” and the opportunity to respond to reviewers’ comments again.

The reviewers provided excellent suggestions for improving the clarity and rigor of our manuscript, which we followed in the enclosed revision. Our responses to reviews are listed below (in blue text), and we highlight major improvements to the manuscript here:

- We removed the use of the term “strain” when referring to Leptospira and incorporated more explanation and context with serogroups.
- We included statistical analyses to support our conclusions.
- We revised the introduction and discussion.

***********************
Reviewer's Responses to Questions

Methods

**Reviewer #1:** This study sought to compare patterns of MAT reactivity among three different wildlife host species infected with *Leptospira interrogans* serovar Pomona. MAT results were also compared across three different laboratories and longitudinally.

*- Are the objectives of the study clearly articulated with a clear testable hypothesis stated*? YES
*- Is the study design appropriate to address the stated objectives?*
In general, the study design was appropriate although the study lacks any use of statistics to compare results. Therefore, it is difficult to know the validity of the conclusions made regarding the existence of differences among groups.
*-Is the population clearly described and appropriate for the hypothesis being tested?* YES.
*-Is the sample size sufficient to ensure adequate power to address the hypothesis being tested?*

For sea lions and foxes, there were a large number of samples available. The small number of skunks (4) limits the power of the study. The lack of statistical analysis makes it difficult to make conclusions about this.
*-Were correct statistical analysis used to support conclusions*? No statistical analysis available

*-Are there concerns about ethical or regulatory requirements being met?* No

Thank you for your thoughtful review of our work. We have modified the manuscript to include group and pairwise statistical comparisons across serovar and host species.

**Reviewer #2:** The purpose of this study is to highlight the limitations of using serology for diseases with high antigenic variability.  The example of MAT and leptospirosis is a notable one because, historically, it has been used to attempt serotyping when no other methods are available.  The data and case studies provided in this study contribute to the already known fact that MAT on serum samples should not be used to identify serovars.  The major issue with the report is that authors elaborate extensively on strain typing which implies a purpose that MAT is not meant to have.  Furthermore, the leptospirosis scientific community has been increasingly recognizing this and promoting a more cautions interpretation.  For example, the MAT panel titers are interpreted at the serogroup level and not serovar.  Even when reporting as the “serogroup with the highest titers”, this should be interpreted in the context of other titers (i.e., >= 4-fold higher), and reported as presumptive serogroup.  Nevertheless, the data reported provide a unique opportunity to show patterns of MAT serogroup titers when there is a known infecting serovar.

Thank you for your comments. We agree that the leptospirosis scientific community has been shifting in its attitudes and practices around the interpretation of MAT data, but in our experience (and in some published works we cite) this practice is not yet universal. Our major intent in writing this manuscript was to support this movement toward more appropriate interpretations, by leveraging the unique dataset of MAT titers from three wildlife species in the context of a known infecting serovar. We have added explicit language describing the more cautious interpretations promoted by the leptospirosis scientific community and have reported the proportion of fox samples that yield an erroneous classification by >= 4-fold titer difference (L245; L393). We have also added explicit information on the serogroups of all serovars reported in our study (L96-98; L133; L179-181), and we note that the results from foxes would yield an incorrect presumptive serogroup as well.

--------------------

Results

**Reviewer #1:**

- *Does the analysis presented match the analysis plan?* The analysis does match the analysis plan.
- *Are the results clearly and completely presented?* At times it is a little difficult to follow the writing and there is the opportunity to simplify the information provided in table format.
- *Are the figures (Tables, Images) of sufficient quality for clarity?* The figures are clear, appropriate, and of sufficient quality. Statistical analysis is needed. Please also include figures that demonstrate the relatedness of the isolates by PFGE.

Thank you for your comments. We have included slightly updated figures for visual cohesiveness, added statistical analyses, and added a PFGE figure (Fig S1) to demonstrate the relatedness of the isolates. We have also striven to make the writing clearer and more concise throughout the manuscript.

**Reviewer #2:**

Line 107: “Positive antibody titers against different strains make assessment of the infecting serovar and identification of epidemiological linkages difficult“.  This is misleading as we know it is not meant to be used for this serovar identification.  It could be rephrased to say that commonly titers are interpreted to determine presumptive serogroup.

This phrase has been deleted from the revised Introduction, and we have been more precise with our wording elsewhere.

Line 110: “complicate any effort to identify the infecting strain from MAT results alone”.  Remove the language referring to “strain” since MAT interpretation is for serogroups.

We made the appropriate change here (L116-117) and have been careful to avoid using the term ‘strain’ for *Leptospira* typing elsewhere in the manuscript as well.

Line 122: “the strain can be typed reliably (or identified as a potential new strain) by PFGE or genome
sequence typing methods, which are much faster and cheaper alternatives to CAAT”.  This is referring to strain typing which has a different dimension and purpose than serotyping. In general, the introduction needs reorganization to focus on the limitations of MAT for serogroup identification.  It is not clear what the authors mean by “strain” in the context of MAT interpretation since they can only refer to serogroups.
We removed the whole paragraph this excerpt was in, and we have refocused and shortened the introduction. As stated above, we have avoided using the term ‘strain’ to refer to *Leptospira* typing.

Line 128:  explanation of serovars and serogroups should be earlier in the introduction.

We have moved the explanation of serovars and serogroups earlier in the introduction and refocused the writing on MAT interpretation for serotyping.

--------------------

Conclusions

**Reviewer #1:**

- *Are the conclusions supported by the data presented?*
- *Are the limitations of analysis clearly described?*
  In general, the data support the conclusions. The conclusions would be greatly strengthened by statistical comparisons. There is no discussion of the reason for not performing a statistical analysis. The authors should also acknowledge the fact that a difference of one serum dilution may not actually be a difference at all (hence the requirement for a 4-fold rise to show a significant difference when doing convalescent testing).
- *Do the authors discuss how these data can be helpful to advance our understanding of the topic under study?* YES
- *Is public health relevance addressed?* YES

We have added language at multiple points in the manuscript (L245; L393) to emphasize the requirement of a 4-fold rise to show a significant difference in titers. We also added this dimension to our results, in interpreting how many fox samples exhibited a higher titer against Autumnalis than against Pomona.

**Reviewer #2:**

Line 326:  “it’s important to recognize that the serovar associated with the maximum titer in a given panel is not necessarily the infecting strain” .  this needs to be interpreted in the context of serogroup.  In the paper in general, using “strain” should be avoided.

We have added the interpretation in terms of serogroups (and note that serovars Autumnalis and Pomona are in different serogroups; L179-180). We have continued to use some serovar language throughout the manuscript, because the MAT panel is defined at that level. We have avoided using the term ‘strain’ to refer to *Leptospira*, but have used it in some more general passages of the manuscript (e.g. the opening paragraphs) because it can apply more generally across many pathogens.

Line 380:  MAT still requires specialized laboratory and trained personnel and it is difficult to access in many parts of the world.

Noted and included (L399-401).
--------------------

Editorial and Data Presentation Modifications?
Use this section for editorial suggestions as well as relatively minor modifications of existing data that would enhance clarity. If the only modifications needed are minor and/or editorial, you may wish to recommend “Minor Revision” or “Accept”.

**Reviewer #1:** The manuscript is reasonably well written, although conciseness could be improved. Some minor errors:
Line 79: “flavivruses” missing an “i”, rickettsia should be rickettsiae
Line 119: with the right conditions, it can be days for culture rather than months
Line 217: consider rewording “PCR- or culture-confirmed skunks” (they were not confirmed as skunks using those methods)
Line 259: labs should be laboratories (also throughout the manuscript)
Line 381: acute and convalescent MAT is considerably more sensitive than organism-detection tests, this is really the main reason for doing it

Thank you for the comments. We have addressed all of the line edits above.

**Reviewer #2:** Citations.  Entire text needs to be checked.  Different parts have different formats.

Thank for you for bringing this to our attention. This was an oversight when we switched citation format in our reference manager prior to submission. All citations should now be updated.

--------------------

Summary and General Comments

Reviewer #1: This was a really interesting study and unique opportunity to examine the utility of MAT for identification of the infecting serovar and highlight the reasons why MAT does not accurately predict the infecting serovar. The inability of MAT to predict the infecting serovar has long been known, but despite this, clinicians and researchers continue to try to use it for this purpose. The primary reason for major revision is the need for statistics to support the conclusion, more data to convince the reader of the relatedness of isolates (PFGE figures), and also to address the length of the manuscript. The introduction and discussion could be shortened substantially (e.g., half the length) as they reiterate what is already widely reported in the literature. I would also recommend including detail in the abstract regarding which wildlife species were included.

Thank you for your comments and feedback. We have included statistical support in the updated version of the manuscript and a PFGE figure (Fig S1). We have also trimmed down the introduction and discussion, removing some entire paragraphs and reorganizing others. While we agree that a lot of the information is widely reported, and we have striven to cite this prior literature as appropriate, we believe it is important to reiterate and synthesize the information in one place. It is our hope that this will help to convince more clinicians and researchers to adopt more robust and modern approaches to type the causative agents of *Leptospira* infections. We have also included the host species in the abstract as you suggest.

Reviewer #2: (No Response)
