# Supplementary material for: Navigating cross-reactivity and host species effects in a serological assay: A case study of the microscopic agglutination test for Leptospira serology
Source: PLoS Negl Trop Dis. 2024 Oct 4;18(10):e0012042. doi: 10.1371/journal.pntd.0012042 (PMC11482713; doi:10.1371/journal.pntd.0012042)
Supplement: Attachment — Submitted filename: Mummah_Navigating cross-reactivity_PLOS_ReviewerResponse2.docx [file pntd.0012042.s003.docx]

**Title:** Navigating cross-reactivity and host species effects in a serological assay: A case study of the microscopic agglutination test for Leptospira serology

**Journal:** PLOS Neglected Tropical Diseases

Dear Editors:

Thank you for the thoughtful reviews of our manuscript titled “Navigating cross-reactivity and host species effects in a serological assay: A case study of the microscopic agglutination test for Leptospira serology” and the opportunity to respond to reviewers’ comments again.

The reviewers provided excellent suggestions for improving the conciseness and clarity of our manuscript, which we followed in the enclosed revision. Our responses to reviews are listed below (in blue text), and we highlight major improvements to the manuscript here:

- We addressed the line edits made by Reviewer 1.

***********************
**Reviewer's Responses to Questions**

**Key Review Criteria Required for Acceptance?**
As you describe the new analyses required for acceptance, please consider the following:

**Methods**
-Are the objectives of the study clearly articulated with a clear testable hypothesis stated?
-Is the study design appropriate to address the stated objectives?
-Is the population clearly described and appropriate for the hypothesis being tested?
-Is the sample size sufficient to ensure adequate power to address the hypothesis being tested?
-Were correct statistical analysis used to support conclusions?
-Are there concerns about ethical or regulatory requirements being met?

Reviewer #1: (No Response)

Reviewer #2: The revised version addressed my concerns.

--------------------

**Results**
-Does the analysis presented match the analysis plan?
-Are the results clearly and completely presented?
-Are the figures (Tables, Images) of sufficient quality for clarity?

Reviewer #1: (No Response)

Reviewer #2: No concerns

--------------------

**Conclusions**
-Are the conclusions supported by the data presented?
-Are the limitations of analysis clearly described?
-Do the authors discuss how these data can be helpful to advance our understanding of the topic under study?
-Is public health relevance addressed?

Reviewer #1: (No Response)

Reviewer #2: The introduction and discussions are much more focused and all the concerns about MAT use and interpretation have been incorporated.

--------------------

**Editorial and Data Presentation Modifications?**
Use  this section for editorial suggestions as well as relatively minor modifications of existing data that would enhance clarity. If the only modifications needed are minor and/or editorial, you may wish to recommend “Minor Revision” or “Accept”.

Reviewer #1: (No Response)

Reviewer #2: (No Response)

--------------------

**Summary and General Comments**
Use  this section to provide overall comments, discuss strengths/weaknesses of the study, novelty, significance, general execution and scholarship. You may also include additional comments for the author, including concerns about dual publication, research ethics, or publication ethics. If requesting major revision, please articulate the new experiments that are needed.

**Reviewer #1:** The authors have improved this manuscript, although I think that there is still room to make it more concise. My line references are for the version of the manuscript with track changes visible (all markup).

Line 170 “reliable…can be achieved using PFGE or WGS” needs a reference. Serovar cannot be directly inferred from PFGE and WGS information, a reference strain library that correlates sequence type back to serovar is required (as in the new figure).

We added a reference as directed and more explicit language about the availability of a reference strain library.

Line 181. For veterinary purposes, often 1 to 15 cultured isolates are used; in human medicine over 20 serovars may be included in MAT (the authors actually state this on line 439)

We updated the text to reflect this information.

Line 200. “quantitative MAT titer levels” – could be just “MAT titers,” also elsewhere in document

We have removed all phrases “quantitative MAT titer levels” and replaced them with “MAT titers” as suggested.

Line 395-396. I think this sentence needs to be deleted as it is redundant.

The line numbering in my versions was a bit messed up (it’s skipping about 100 lines in the middle and looks the same when I download the version from the editorial manager) and didn’t make these references exactly. This was the only line reference where I had to guess a bit. I believe the reviewer is referring to the last line of the first paragraph of the discussion. We have removed this sentence (“We outline lessons…interpreting MAT results.).

Line 443. “At the bottom line…” This sentence is not concise and could even be deleted.

We shortened this sentence but did not remove it.

Line 543. “Genetic methods remain superior to serology for serotyping…” does not make sense. By definition, serotyping is a serologic technique. The benefit of MAT is that it is more sensitive for diagnosis that organism-detection tests such as PCR and culture; this needs to be stated.

We have removed the reference to serotyping and now focus only on the benefits of MAT. We also mention the increased sensitivity of MAT over culture-based methods.

The new supplemental figure improves the manuscript.

Reviewer #2: (No Response)

--------------------

PLOS authors have the option to publish the peer review history of their article (<a href="<https://journals.plos.org/plosntds/s/editorial-and-peer-review-process#loc-peer-review-history>" target="_blank">what does this mean?). If published, this will include your full peer review and any attached files.

If you choose “no”, your identity will remain anonymous but your review may still be made public.

Do you want your identity to be public for this peer review? For information about this choice, including consent withdrawal, please see our <a href="<https://www.plos.org/privacy-policy>" target="_blank">Privacy Policy.

Reviewer #1: No

Reviewer #2: No
